# Immune Complex Glomerulonephritis Following Bone Marrow Transplantation in C3 Deficient Mice

**DOI:** 10.1371/journal.pone.0003334

**Published:** 2008-10-06

**Authors:** Thomas R. Welch, Lisa W. Blystone

**Affiliations:** Department of Pediatrics, SUNY Upstate Medical University, Syracuse, New York, United States of America; Instituto Oswaldo Cruz and FIOCRUZ, Brazil

## Abstract

**Background:**

The role of circulating complement in host defense and immune disease is well established. Although a number of cells and tissues are capable of synthesizing complement components locally, the importance of such local synthesis in immune disease has been difficult to establish.

**Methodology/Principal Findings:**

We used bone marrow transplantation (BMT) between C3 knockout (C3KO) and wild type (WT) mice to construct animals that were discordant for systemic (hepatic) and local (monocytic) C3 synthetic capacity. An immune complex glomerulonephritis (GN) was then induced using intraperitoneal injections of horse spleen apoferritin (HSA) with a lipopolysaccharide (LPS) adjuvant. All HSA/LPS animals developed a proliferative GN with glomerular infiltration by monocytes. By sensitive ELISA, monocyte C3 synthesis could be detected in C3KO animals transplanted with WT bone marrow cells. Despite this, there were no significant differences among groups of mice in measures of clinical (proteinuria, renal function) or histologic (glomerular cellularity, crescents) disease severity.

**Conclusions/Significance:**

In this model of GN, local synthesis of C3 by infiltrating cells does not appear to be of pathologic importance.

## Introduction

The complement proteins are major effectors of inflammation in glomerulonephritis, both in humans and in animal models. The bulk of complement deposited in the glomerulus in acute glomerulonephritis is presumed to come from the circulation, and is synthesized in the liver. This constitutes “systemic” complement synthesis.

A variety of other cells, however, are capable of synthesizing complement components. Most of these are epithelial; we have shown that renal proximal tubule epithelial cells (PTEC) synthesize a variety of components in humans and mice [1]–[3]. There is also good evidence that hematopoietic cells of the monocyte/macrophage lineage synthesize a variety of complement components, both in vivo and in vitro [4]. Recent work has suggested that monocyte-derived C3 may be important in the local immune response in the reticuloendothelial system [5]. This, in turn, constitutes “local” complement synthesis.

Many glomerulopathies are characterized by a mononuclear cell infiltrate. It has been suggested that complement synthesized by these infiltrating cells could be an important mediator of glomerular injury [6].

Although we have reported several studies addressing the role of locally synthesized complement by tubular epithelium in the renal interstitium [1]–[3], [7], the role of monocyte-derived complement in the glomerulus has not been addressed. In the experiments reported herein, we employ a novel strategy by which animals deficient in C3 undergo hematologic reconstitution with bone marrow from C3 replete animals. This strategy makes it possible to separate the contribution of systemic (liver-derived) complement from that potentially produced by monocytes.

## Materials and Methods

### Experimental Animals

C57BL/6 mice, either wild type (WT) or homozygous C3 knockout (C3KO), were purchased from The Jackson Labs (Bar Harbor, ME). The protocol was reviewed and approved by the Committee for the Humane Use of Animals at SUNY Upstate Medical University.

### Creation of chimeras by bone marrow cell (BMC) transplantation

Eight week old male mice were irradiated with 11 Gray, a lethal dose which is split into two half doses delivered four hours apart. Such a dose regime avoids a substantial amount of gut induced radiation disease, and minimizes gastric distress. To prevent death from nasal pseudomonas, twenty-four hours prior to, and for one week after irradiation, the animals were given tetracycline (6.5 mg/ml terramycin in acidified water, administered ad libitum). On the day following irradiation, each animal received approximately four million bone marrow cells (BMC) that had been harvested by flushing the marrow cavity of femurs and tibiae of donor WT or C3KO mice with DMEM (Invitrogen, Carlsbad, CA) supplemented with 1% fetal calf serum (FCS; Hyclone, Logan, UT). The BMC were washed several times with PBS and then contaminating red blood cells were lysed with AKC buffer (0.15 M NH_4_CL, 10 mM KHCO_3_ and 0.1 mM EDTA, pH 7.35). The BMC were washed, resuspended in DMEM, counted, and injected intravenously via a tail vein using a 26-gauge needle.

The chimeras created by this process were defined as follows:

WT^IR^/WT–WT background irradiated, reconstituted with WT bone marrow cells
WT^IR^/C3KO–WT background irradiated, reconstituted with C3KO bone marrow cells
C3KO^IR^/WT–C3KO background irradiated, reconstituted with WT bone marrow cells
C3KO^IR^/C3KO–C3KO background irradiated, reconstituted with C3KO bone marrow cells

The first and fourth groups served as controls for the processes of irradiation and reconstitution.

### Induction of glomerulonephritis

The irradiated and transplanted animals were allowed to recover for 4 weeks, at which time immune complex glomerulonephritis was induced using a method we have described previously [3]. Briefly, 10 mg horse spleen apoferritin (HSA; Sigma, St. Louis) were injected intraperitoneally (IP) five days per week. One hundred micrograms lipopolysaccharide (LPS; from *Salmonella minnesota*; EMD Biosciences, La Jolla, CA) were delivered by the same route three days per week as adjuvant. Each group of transplanted animals was composed of six male mice who were approximately 12 weeks old at the time of induction of glomerulonephritis. Additional groups of six WT and six C3KO animals (also 12 weeks old ) served as vehicle control groups and received 0.2 ml injections of 150 mM NaCl, IP, five days per week. All six groups of mice were injected by this protocol for a total of six weeks.

### Harvesting of tissues

Nine weeks after starting injection, animals were euthanized by CO_2_ narcosis. For 24 hours prior to euthanasia, they were placed in metabolic cages for collection of urine. Immediately after death, blood was obtained by vena cava puncture, and tissue was obtained from kidney and spleen. Some tissue samples were place in 10% buffered formalin. American Histolabs, Inc., (Gaithersburg, MD), performed embedding, sectioning and staining with either hematoxylin and eosin, or periodic acid-Schiff (PAS) stain for histology. Additional tissue samples were quick frozen in M-1 embedding matrix (Thermo Shandon, Pittsburgh, PA) for immunohistochemistry.

### Histological analysis of kidney sections

A series of digital images representing the complete cortex from a periodic acid-Schiff-stained kidney section from each animal was collected with a SPOT Diagnostics camera (Sterling Heights, MI), as we have previously described [7]. These images were then analyzed for the following measures:

▪ Glomerular cellularity, defined as the total cells in ten representative glomerular cross sections.
▪ Glomerular crescents, defined as two or more cell layers surrounding the glomerular tuft, in 50 glomeruli.

### Immunohistochemistry of kidney sections

Frozen sections of kidneys were thawed slowly and washed with PBS, followed by incubation with either 10% normal goat serum (for C3c slides) or 10% normal rat serum (for CD14 slides) for 30 minutes at 37°C. Excess serum was blotted and the slides were incubated for one hour at 37°C with either goat anti-mouse C3c with a FITC label (Nordic Immunology/Accurate Chemical, Westbury, NY), or rat anti-mouse CD14 (eBioscience, San Diego, CA), both antibodies diluted in PBS. Slides were again washed with PBS, and the CD14 slides were incubated with goat anti-rat IgG–FITC (Jackson ImmunoResearch, West Grove, PA) for an additional 30 minutes at 37°C. Following incubation with antibodies, slides were washed with PBS and then mounted with 0.1% *o*-phenylenediamine in PBS-glycerol.

### Plasma C3 concentration

The concentration of C3 in the plasma was measured by Mouse High-Sensitive Complement C3 ELISA (Kamiya Biomedical Company, Seattle, WA), following manufacturer's directions. For this double antibody sandwich ELISA, samples from WT background mice were diluted 1∶50,000, and samples from C3KO background mice were diluted 1∶50.

## Results

### Chimeras

**Figure 1:**
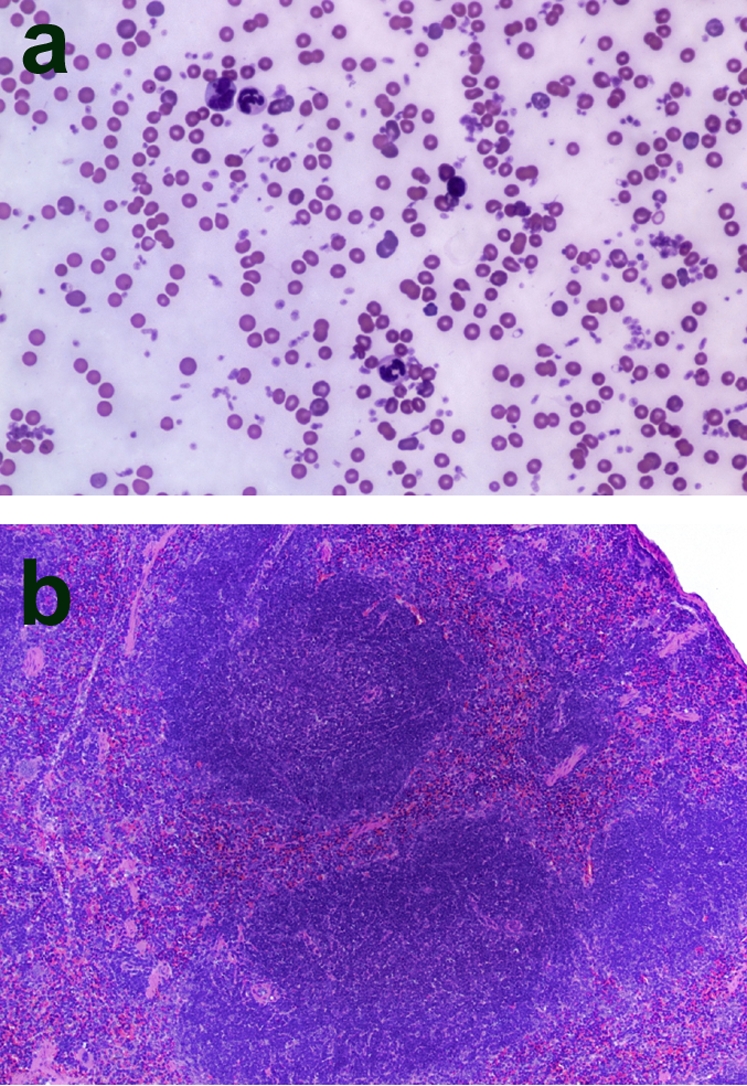
Confirmation of hematologic reconstitution of mice that were lethally irradiated and then transplanted with donor bone marrow cells; magnification, ×100. a: Wright-Giemsa stained peripheral blood smear from a C3KO animal 14 weeks after bone marrow transplant from a WT mouse. Note presence of all three cell lines (erythrocytes, leukocytes, platelets). b: Hematoxylin and eosin stained spleen section from a C3KO mouse 14 weeks after transplantation from a WT animal. Note normal appearing lymphatic nodules.

The bone marrow transplantation process created chimeras that survived to undergo the injection protocol. Examination of peripheral blood smears and reticuloendothelial structures (spleen) of selected animals confirmed hematologic reconstitution (Figure 1).

### Clinical disease

All animals receiving HSA/LPS developed proteinuria (Table 1). There were no significant differences in the quantity of urine protein among the four groups of chimeras. There were also no significant differences among the chimera groups in renal function, as assessed by measurements of blood urea nitrogen, and plasma albumin and creatinine, at the time of harvest (Table 1).

**Table 1:** Comparison of the degree of proteinuria and the status of renal function following induction of glomerulonephritis in chimeric mice versus saline injected controls.

	Urine Albumin (µg/ml)	Blood Urea Nitrogen (mg/dl)	Plasma Creatinine (mg/dl)	Plasma Albumin (g/dl)
WT-Saline	24.5±7.9^a^	23.5±1.2	0.27±0.02	2.78±0.18
C3KO-Saline	47.2±9.9+	27.2±1.9	0.22±0.02	3.07±0.26
WT^IR^/WT	604.5±157.9	53.7±3.4	0.43±0.02	2.55±0.03
WT^IR^/C3KO	604.2±170.2	49.7±12.9	0.40±0.05	2.50±0.10
C3KO^IR^/WT	599.5±277.0	38.8±6.8	0.32±0.04	2.33±0.28
C3KO^IR^/C3KO	613.7±145.9	92.0±36.29	0.45±0.15	2.63±0.13

a Mean±S.E.M.

+ P = 0.102 vs. WT-Saline.

### Glomerular histology and immunohistochemistry

**Figure 2:**
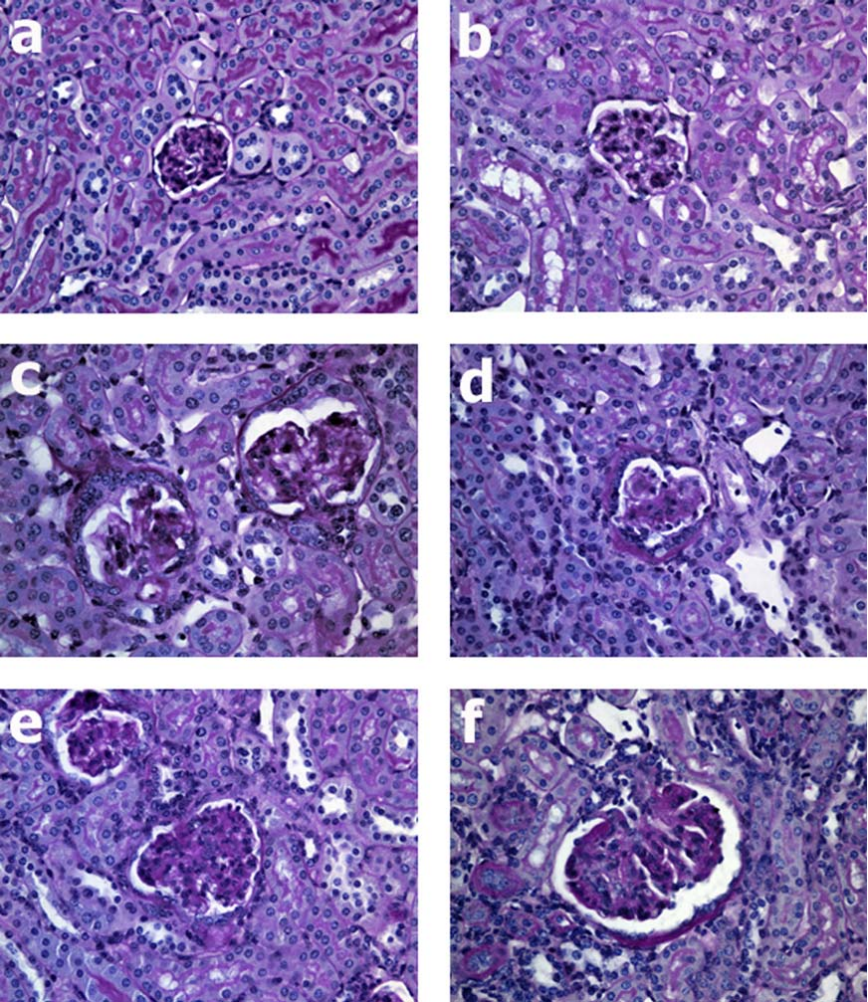
Histology of kidney sections. All sections were stained with Periodic Acid-Schiff stain; magnification ×400. a: WT mouse treated with saline. b: C3KO mouse treated with saline. c: WT^IR^/WT mouse treated with HSA/LPS. d: C3KO^IR^/WT treated with HSA/LPS. e: WT^IR^/C3KO mouse treated with HSA/LPS. f: C3KO^IR^/C3KO treated with HSA/LPS.

**Figure 3:**
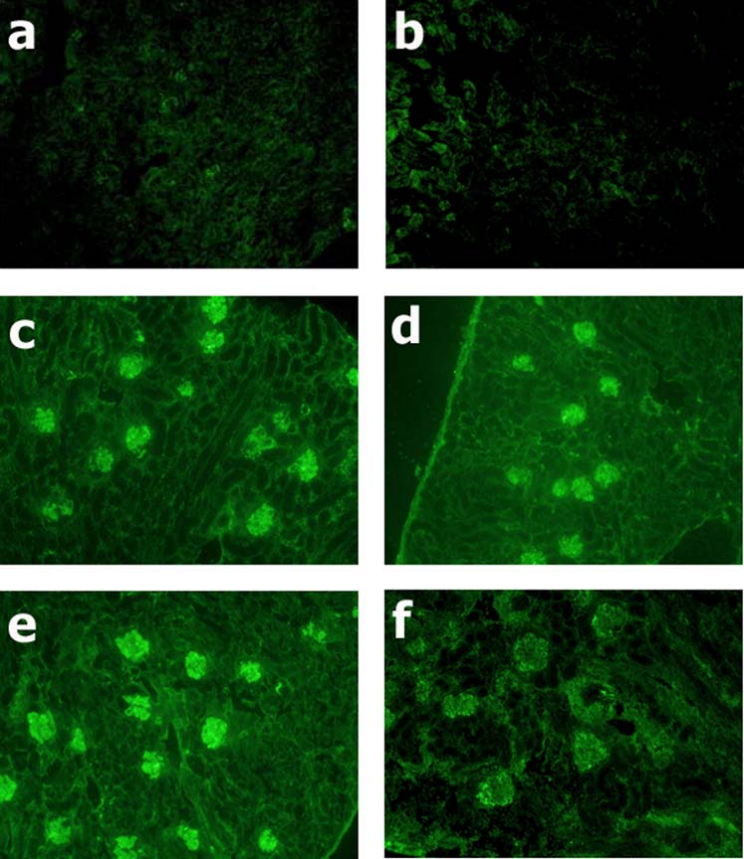
Immunohistochemical localization of CD14. Sections were stained with rat anti-mouse CD14 followed by goat anti-rat IgG labeled with FITC; magnification ×100. a: WT mouse treated with saline. b: C3KO mouse treated with saline. c: WT^IR^/WT mouse treated with HSA/LPS. d: C3KO^IR^/WT mouse treated with HSA/LPS. e: WT^IR^/C3KO mouse treated with HSA/LPS. f: C3KO^IR^/C3KO mouse treated with HSA/LPS.

**Figure 4:**
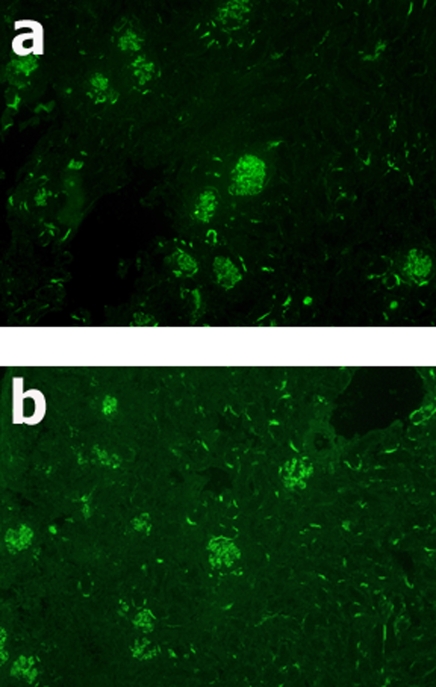
Immunohistochemical localization of C3 in WT background chimeric mice treated with HSA/LPS. 100× views of kidney sections from WT^IR^/WT (a) and WT^IR^/C3KO (b) chimeras stained with FITC-conjugated goat anti-mouse C3c.

All animals receiving HSA/LPS injections developed a proliferative glomerulonephritis (HSA/LPS treated mice are shown in Figure 2c–f) as compared to a WT and C3KO mouse treated with saline shown in Figure 2a–b. FITC-conjugated anti-CD14 staining demonstrated that the predominate cell type infiltrating the glomeruli was monocytes (Figure 3c–f). Background staining of CD14 in either a WT or a C3KO mouse treated with saline is shown is Figure 3a–b. As we have shown previously in intact animals [3], there was evidence of C3 glomerular deposition in each chimera with a wild type background (WT^IR^/WT and WT^IR^/C3KO; Figure 4). As objective measures of the severity of glomerular inflammation, glomerular cell counts and crescent formation were determined for each animal, and among group comparisons made. There were no differences in either measure of nephritic severity among the chimera groups (Table 2).

**Table 2:** Comparison of glomerular cell counts and crescent formation in chimeric mice treated with HSA/LPS versus saline injected controls.

	Glomerular Cells^b^	Glomerular Crescents^c^
WT-Saline	28.9±1.0^d^	0
C3KO-Saline	30.5±0.7	0
WT^IR^/WT	52.2±3.7	14.0±0.8
WT^IR^/C3KO	45.3±1.4	11.0±1.4
C3KO^IR^/WT	49.6±2.1	11.5±1.1
C3KO^IR^/C3KO	57.3±2.4	14.7±0.7

b Cells per 10 glomeruli.

c Crescents per 50 glomeruli.

d Mean±S.E.M.

### Plasma C3 concentration

**Figure 5:**
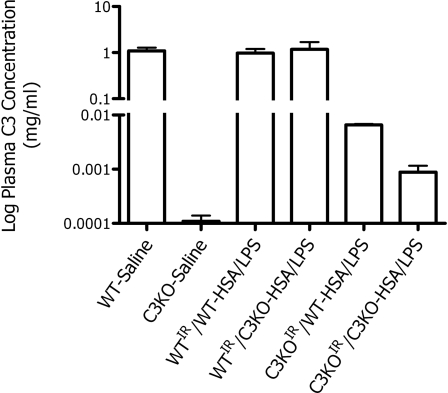
Plasma C3 concentrations as determined by ELISA. Measurements are from terminal plasma samples and demonstrate normal levels of C3 in all WT mice. C3KO animals and C3KO^IR^/C3KO chimeras had C3 concentrations below 0.001 mg/ml, approaching the lower limit of the assay. However, there was measurable C3 in the plasma of C3KO^IR^/WT chimeric mice.

Plasma C3 concentrations in the experimental animals are shown in Figure 5. There were no significant differences in concentration between the chimeras with WT backgrounds (WT^IR^/C3KO and WT^IR^/WT). On the other hand, when C3KO animals were reconstituted with WT bone marrow cells, detectable C3 was present in plasma, albeit at only a fraction (0.67%) of that found in WT animals. C3KO animals and C3KO^IR^/C3KO chimeras had C3 concentrations below 0.001 mg/ml, approaching the lower limit of the assay.

## Discussion

Several lines of evidence support a role for the complement system in many forms of glomerulonephritis [8]. Complement components can be shown to be deposited within affected glomeruli. The concentration of many complement components in plasma is reduced during active glomerulonephritis, and circulating complement component breakdown products can be detected.

The liver is the source of most circulating complement. For several years, however, it has been appreciated that there is extrahepatic synthesis of a variety of components, both in humans and in experimental animals [9]. The in vivo importance of this, however, remains unclear. In a model of murine herpes virus infection, for example, local complement synthesis and activation in the myeloid compartment has been shown to enhance the development of humoral immunity [5]. This effect may be mediated via C3a binding to bone marrow dendritic cells, since inhibition of such binding impairs T cells response to antigen presented by those cells 10]. In vitro, PTEC with bound C3b enhanced alloreactive T cell activation [11].

A number of published studies of extrahepatic complement synthesis have dealt with the kidney and monocytes. Renal complement synthesis was first demonstrated in lupus-prone (NZB) mice by Passwell and colleagues [9]. Subsequently, our laboratory established that the renal proximal tubular epithelium was the source of kidney mRNA for complement components, in both humans and mice [1]–[3]. This observation paralleled other studies of renal tubular epithelial cells in vitro [12], [13].

The role of this tubular complement synthesis has been examined in experimental animals, based upon observations in human disease. Chronic glomerulonephritis that progresses to ESRD is always accompanied by tubulointerstitial disease: peritubular edema, fibrosis, inflammatory cell infiltration, and tubular atrophy. We have employed experimental animals models to support an important role for such tubular complement component synthesis in tubulointerstitial disease [3], [14]. There is also experimental evidence of a role for local renal C3 synthesis in reperfusion injury [15]. Alternative pathway complement activation and occupation of the C3a receptor on proximal tubular epithelial cells promotes cytokine expression in a murine reperfusion model [16].

The in vivo role of monocyte-derived complement, specifically C3, has been studied much less. Studies in C3KO^IR^/WT chimeras, similar to those we have employed, have suggested a role for local (presumably monocyte/macrophage-derived) C3 in the reticuloendothelial system in the immune response to viruses [5]. This study, as is the case in ours, showed evidence of some low-level C3 present in plasma as well. Conversely, other studies have suggested a role for C3 in the differentiation of monocytes into dendritic cells [17].

The synthesis of monocyte C3 and other complement proteins could theoretically be important in the glomerular component of glomerulonephritis. Many glomerulonephritides, both in humans and in animal models, are characterized by monocytic infiltrate. In vitro, monocyte C3 synthesis is enhanced by stimuli such as cytokines [4] and immune complexes [6]. Obviously, both of these stimuli may be in abundance in glomeruli affected by immune complex glomerulonephritis. Thus, it is reasonable to hypothesize that infiltrating monocytes could amplify the inflammatory process by providing a source of local C3.

Testing this hypothesis in intact animals would be difficult. The presence of circulating liver-derived C3 in plasma would make it impossible to establish the presence of what would likely be quantitatively smaller amounts of locally synthesized protein. Moreover, the known strong in situ hybridization signal from tubular C3 mRNA could overwhelm a weaker glomerular signal [3].

To overcome this problem, we employed the construction of “chimeras”, similar to work done by Verschoor et al. who studied the immune response to herpes virus [5]. This strategy permitted us to induce immune complex glomerulonephritis in animals lacking normal systemic C3 synthetic capability, but with a hematopoietic system reconstituted from wild type mice.

As we have noted previously, the glomerulonephritis model we have employed resulted in marked glomerular hypercellularity. Using immunofluorescent techniques, we demonstrated that much of this hypercellularity is derived from infiltrating monocytes. Additionally, animals with wild type background had evidence of C3 deposition in their glomeruli. Our previous studies have also shown such deposits of C3 in the glomerulus, beginning at about three weeks after induction of glomerulonephritis [3]. Although some plasma C3 could be detected in C3KO^IR^/WT chimeric mice, they did not have immunohistochemical evidence of glomerular C3 deposition.

As would be expected from the work of Drouin and colleagues [4], monocytes from these animals should express C3 mRNA following cytokine stimulation. The presence of detectable C3 protein by ELISA in the plasma collected from our C3KO^IR^/WT chimeric mice confirms this, although the concentration is unlikely to be physiologically important. Some workers have suggested a role for C3 synthesis by monocytes in local inflammatory conditions such as glomerulonephritis and transplant rejection [18], [19]. Two lines of evidence in the studies reported herein suggest that such an effect is either nonexistent or trivial.

First of all, immunohistochemistry for C3 in C3KO animals reconstituted with WT bone marrow cells shows no C3 glomerular deposition, despite active inflammation and trace C3 protein in infiltrating cells. Such experiments are critical to establishing this, since the strong C3 deposition in C3 replete (i.e. WT) mice would overcome any locally synthesized proteins.

The other important observations relate to the severity of the histologic and clinical disease. There was no difference in two histologic markers of severity (glomerular cell counts and crescents) between C3KO and WT animals, regardless of whether they were reconstituted with WT or C3KO bone marrow cells. Furthermore, there was no difference among groups in proteinuria or renal function. The glomerular histology in these animals did not differ from our previously published observations in untransplanted animals [7].

It could be argued that, despite glomerular deposition in WT animals, C3 is not critical to the glomerular component of this model. As our previous studies have shown [3], a variety of inflammatory mediators are likely present in these affected glomeruli; these are clearly contributing to an exuberant disease regardless of the presence or absence of C3. Indeed, the severity of the glomerular lesion appears to be independent of the presence or absence of systemic C3 synthetic capacity.

The rationale of these studies, however, was to develop a model that would permit differentiating the effect of systemic synthesis, activation and deposition of a complement component from that which is produced locally, in much smaller quantities and mediates its effects in the immediate microenvironment. Thus, our results suggest that in our specific model of immune complex glomerulonephritis, neither systemic nor locally synthesized C3 is critical to the glomerular inflammatory process. A similar bone marrow transplant strategy could be employed in other models in which the glomerular role of C3 appeared more critical.

Finally, it should be noted that the focus of these experiments was exclusively on glomerular and monocytic C3. Our previous studies have suggested that the importance of locally synthesized complement is most likely in the tubulointerstitial component of inflammatory renal disease, where it is synthesized by the renal tubular epithelium [3].

These studies demonstrate the importance of examining intact animal models of disease, in addition to in vitro work, when considering the role of locally synthesized inflammatory mediators. Although monocytes clearly are capable of synthesizing the central protein of the complement cascade in response to inflammation, this ability does not appear to have an important role in vivo.
